# Biofeedback Impact on Limb Stiffness and Joint Power in Patients With ACL Reconstruction—A Secondary Analysis

**DOI:** 10.1002/jor.70131

**Published:** 2025-12-31

**Authors:** Vaibhavi Rathod, Bryana N. Vasquez, Michael A. Teater, Sara L. Arena, Robin M. Queen

**Affiliations:** ^1^ Granata Biomechanics Lab, Department of Biomedical Engineering and Mechanics Virginia Tech Blacksburg Virginia USA; ^2^ Department of Orthopaedic Surgery Virginia Tech Carilion School of Medicine Roanoke Virginia USA

**Keywords:** biomechanics, eccentric training, normalized symmetry index, physical therapy, rehabilitation

## Abstract

The purpose of the study was to examine the effects of a biofeedback intervention on limb stiffness and eccentric knee joint power (ECCKP) symmetry, as well as related landing biomechanics, in individuals post‐anterior cruciate ligament reconstruction (ACLR). Thirty‐three participants (Biofeedback: *n* = 14; Control: *n *= 19) completed a 12‐week protocol with assessments at baseline, post‐intervention, and retention time‐points. Linear mixed‐effects models evaluated the effects of group and time‐point on ECCKP normalized symmetry index (NSI) and limb stiffness NSI. Linear mixed‐effects models evaluated the effects of group, time‐point, and limb (surgical vs. non‐surgical) on limb stiffness, peak relative ground reaction force (rGRF), change in limb length, and time to peak rGRF during landing. There were no significant group × time‐point interactions found for ECCKP NSI or limb stiffness NSI (*p* > 0.05). For limb stiffness, the non‐surgical limb showed greater stiffness (*p* = 0.001). Peak rGRF was higher in the non‐surgical limb (*p* = 0.001) and at baseline compared to post‐intervention (*p* = 0.023). Time to peak rGRF was longer in the non‐surgical limb (*p* = 0.008). No significant effects were found for change in limb length. Overall, the biofeedback intervention did not significantly improve ECCKP or limb stiffness symmetry post‐ACLR. Persistent symmetry deficits in landing biomechanics were evident, particularly between surgical and non‐surgical limbs. Given that the study was likely underpowered to detect moderate effects, findings should be interpreted with caution. Larger, adequately powered studies are warranted to further evaluate the effect of biofeedback in improving symmetry during dynamic tasks after ACLR.

**Trial Registration:** ClinicalTrials.gov: AR069865.

## 1 Introduction

Approximately one in four adolescent athletes who undergo anterior cruciate ligament reconstruction (ACLR) and return to sport (RTS) sustain a second ACL injury, often occurring during an early exposure after returning to sports [1, 2, 3, 4, 5]. These athletes typically participate in high‐risk sports involving cutting, jumping, and landing [6, 7]. Despite successful surgical outcomes, only 55% of patients following ACLR return to their pre‐injury competitive level [4, 8] and many continue to exhibit neuromuscular deficits upon RTS [1, 2, 3].

RTS clearance is traditionally guided by a combination of patient‐reported outcome measures (PROMs) and physical performance tests that evaluate perceived function and limb symmetry [9]. PROMs such as the International Knee Documentation Committee (IKDC) score, Knee Injury and Osteoarthritis Outcome Score (KOOS), and Lysholm scale are commonly employed to assess self‐perceived recovery [10, 11, 12]. Functional tests, including hop tests and assessments of isokinetic quadriceps strength are frequently used to evaluate physical readiness [13]. These assessments are typically administered by physical therapists collaboratively with sports medicine physicians or orthopedic surgeons. However, these measures often emphasize task completion rather than movement quality, and their thresholds for RTS (e.g., ≥ 90% interlimb symmetry [8, 14]) lack universal validation [13, 15, 16]. Consequently, many athletes achieve “passing” scores despite persistent deficits in neuromuscular control and limb loading symmetry [17]. Impaired neuromuscular control and inter‐limb symmetry compromise eccentric control during deceleration and may underlie the high rates of graft failure and contralateral injuries observed post‐RTS.

Eccentric knee joint power (ECCKP) and limb stiffness play crucial roles in determining an athlete's ability to safely RTS [18, 19]. ECCKP represents the knee's ability to store energy during deceleration movements that are fundamental to both athletic performance and injury prevention [20]. Following ACLR, many athletes experience quadriceps atrophy and strength deficits resulting in impaired ECCKP, limiting the limb's ability to absorb impact forces and increasing reliance on the contralateral limb during landing [21]. Orishimo et al. reported a 43% reduction in ECCKP absorption in the surgical limb 7 months post‐ACLR, suggesting incomplete recovery which may lead to prolonged neuromechanical deficits following ACLR [22]. Impaired ECCKP following ACLR not only limits performance but also increases strain on the ACL graft, increasing re‐injury risk [23, 24, 25, 26, 27].

Altered limb stiffness, quantified as the ratio of peak resultant ground reaction force (peak rGRF) to the change in limb length as a potential risk factor for second ACL injuries [28, 29]. Post‐ACLR athletes often exhibit increased limb stiffness due to quadriceps weakness, scar tissue formation, and reduced range of motion, leading to asymmetrical loading and impaired landing mechanics [30]. Increased limb stiffness during the initial 100 ms of ground contact, impairs landing mechanics and increases ACL loading, further increasing re‐injury risk [20, 31]. Rehabilitation programs should therefore target both ECCKP and limb stiffness to optimize recovery and reduce re‐injury risk.

Eccentric training is an effective intervention for improving biomechanical factors related to second ACLR injury risk [32, 33, 34]. However, traditional rehabilitation often fails to provide real‐time feedback, limiting the ability to adjust movement patterns. Biofeedback‐based interventions provide real‐time feedback on neuromuscular activity, movement patterns, and force distribution [35, 36]. Previous studies show that visual electromyographic (EMG) biofeedback improves knee extension strength and proprioception in ACLR patients [37, 38]. Similarly, tactile biofeedback, such as resistance band training, improves knee moment symmetry [39]. However, most existing protocols focus on single‐modality feedback and isolated strength improvements rather than integrated movement strategies. The combined effect of visual and tactile biofeedback on these dynamic, performance‐relevant parameters remains unknown. The purpose of this pilot study was to investigate the effect of a combined visual and tactile biofeedback intervention on interlimb symmetry in ECCKP and limb stiffness among athletes following ACLR. We hypothesize that participants receiving biofeedback would demonstrate improved ECCKP and limb stiffness symmetry compared to controls. This study aims to inform the development of rehabilitation strategies that move beyond strength restoration toward functional, data‐driven readiness for RTS. The study was not powered to detect small or moderate between‐group differences, the goal of this pilot trial was to assess feasibility and generate preliminary data to inform future investigations.

## 2 Methods

The study is a secondary analysis from the ACL‐Biofeedback Trial, an assessor‐blinded, randomized, controlled trial (ClinicalTrials.gov: AR069865) [40]. The study was approved by the Institutional Review board (IRB #17‐007) and all patients provided written informed consent. Participant recruitment, eligibility criteria, and detailed descriptions of the biomechanical assessments, biofeedback intervention, and control condition are outlined in Queen et al. [40] Forty participants who underwent primary unilateral ACL reconstruction (ACLR) using either bone‐patellar tendon bone (*n* = 20) or hamstring autografts (*n* = 20) were recruited from affiliated orthopedic clinics. All surgeries were performed by experienced orthopedic surgeons following standardized anatomical single‐bundle reconstruction techniques. Surgical consistency across participants was verified through operative reports. Among these participants, 12 underwent concomitant meniscal repair and 9 underwent partial meniscectomy.

Participants were randomized in a 1:1 ratio to either the biofeedback (BF) or control (C) group using block randomization, with block size unknown to investigators and study staff to ensure allocation concealment. Randomization was stratified by gender, age (younger: 14–17 years; older: 18–21 years), and activity level (mild: Tegner 1–3; moderate: Tegner 4–7; vigorous: Tegner 8–11). Eligible participants provided informed consent and completed baseline biomechanical testing prior to randomization. Group assignment was revealed at the first study visit (either biofeedback intervention or control education session). The randomization sequence was computer‐generated by the project statistician and managed through the REDCap randomization module [41, 42].

The BF group completed supervised, twice‐weekly sessions for 6 weeks focusing on restoring symmetrical loading and frontal plane knee joint position during squatting. Each session included visual and tactile feedback components. Real‐time visual feedback was provided via a monitor displaying vertical ground reaction forces beneath each foot as a bar graph, allowing participants to adjust movement to maintain load symmetry (LSI ≥ 90%). Tactile feedback was delivered using a resistance band positioned on the surgical limb, pulled at approximately 45° angle toward the contralateral side. This setup required participants to actively resist frontal plane valgus and promote equal weight bearing. A handheld dynamometer (Rolyan Smart Handle, Performance Health, Warrenville, IL) was used to maintain a consistent load across trials and sessions, determined by the clinician's judgment. Each session included 30 visual feedback squats and 30 tactile feedback squats (3 sets of 10 repetitions each), performed at a standardized pace. The control group completed a 6‐week attention control program focusing on educational modules on ACLR rehabilitation milestones, RTS criteria, and injury prevention principles, but did not include any movement‐based feedback or supervised exercise.

**Figure 1:**
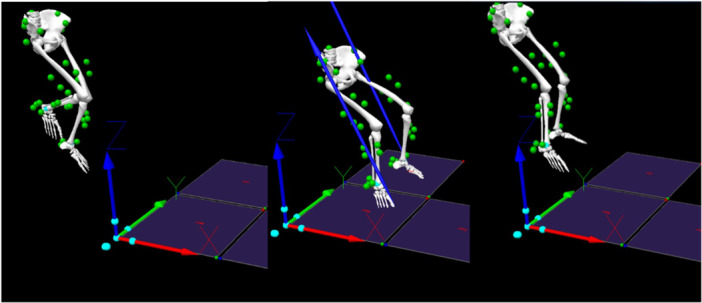
First landing of stop‐jump task in Visual 3D (take‐off, approach, and landing; Blue arrows: GRF).

Participants completed three biomechanical assessments at baseline, post‐intervention (6 weeks), and retention visit (12 weeks). The primary assessment involved a stop‐jump (SJ) task, selected for its ability to replicate sport‐like movements such as a basketball jump shot or soccer header [43]. Three‐dimensional kinematics were captured using a 10 camera motion capture system (Qualisys, Gothenburg, Sweden) sampling at 240 Hz, with 43 retroreflective markers placed according to the modified Helen‐Hayes configuration [44]. Ground reaction forces were simultaneously collected using embedded force plates (AMTI, Watertown, MA) at 1920 Hz (Figure 1).

### 2.1 Data Processing

The kinematic and kinetic data were filtered with a fourth‐order low‐pass Butterworth filter, using cutoff frequencies of 7 and 100 Hz, respectively. Details of signal processing and inverse dynamics calculations are described in Queen et al. [40]. However, a concise summary is provided here for clarity.

All data were extracted from the first bilateral landing of the SJ task. The landing phase of the SJ was defined as the portion of ground contact that began at initial contact (vGRF > 10 N) and ended at the maximum inferior displacement of the sacral marker [45]. ECCKP was calculated for the entire landing phase to report how much energy the knee extensors stored during landing. ECCKP was normalized by body weight and was determined by finding the minimum value which corresponded to the peak eccentric power occurring at the knee joint during landing. Limb stiffness was evaluated during the first 100 ms after initial contact [46]. A 3D vector was defined from the hip joint center (HJC) to the center of pressure (COP) of the ipsilateral limb to represent the limb [28]. The resultant GRF (rGRF) vector was projected onto the limb vector by calculating a dot product of the GRF and limb vectors. To accommodate for the various speeds at initial contact caused by takeoff distance and jump height, peak rGRF was normalized by resultant kinetic energy at initial contact [47]. Change in limb length was defined as the difference between limb length at initial contact and limb length at the peak rGRF. Change in limb length was normalized by initial limb length. Limb stiffness was calculated using the ratio of peak rGRF and the change in the limb length vector (Equation 1) [28].

(1) $$K_{\mathrm{limb}}=\frac{\mathrm{Peak}\;rG\mathrm{RF}}{∆\mathrm{Limb}\;\mathrm{Length}}$$

The absolute value of limb stiffness and the ECCKP were used to calculate limb stiffness (Equation 2) and ECCKP symmetry (Equation 3). The NSI ranges from 0%, indicating full symmetry to 100%, indicating full asymmetry. The NSI is designed for variables measured across multiple trials (at least 3) [48], with a single trial denoted as *t*. In this study, participants performed 10 trials of the SJ tasks [48].

(2) $$K_{\mathrm{limb}}\mathrm{NSI}=\;\left|\frac{K_{\mathrm{limb},\mathrm{NS},t}-K_{\mathrm{limb},S,t}}{\max_{t=1:n}\left(\max\left(0,K_{\mathrm{limb},\mathrm{NS},t},K_{\mathrm{limb},S,t}\right)\right)-\min_{t=1:n}\left(\min\left(0,K_{\mathrm{limb},\mathrm{NS},t},K_{\mathrm{limb},S,t}\right)\right)}\times 100\right|$$

(3) $$\mathrm{ECCKP}\;\mathrm{NSI}=\;\left|\frac{{\mathrm{ECCKP}}_{\mathrm{NS},t}-{\mathrm{ECCKP}}_{S,t}}{\max_{t=1:n}\left(\max\left(0,{\mathrm{ECCKP}}_{\mathrm{NS},t},{\mathrm{ECCKP}}_{S,t}\right)\right)-\min_{t=1:n}\left(\min\left(0,{\mathrm{ECCKP}}_{\mathrm{NS},t},{\mathrm{ECCKP}}_{S,t}\right)\right)}\times 100\right|$$

### 2.2 Statistical Analysis

Linear mixed‐effects models were employed to examine the effects of treatment group (Biofeedback vs. Control) and time‐point (Pre vs. Post vs. Ret) on two primary symmetry outcomes, ECCKP and limb stiffness symmetry. For these models, treatment group and time‐point were included as fixed effects, and participant was included as a random effect.

In addition to the symmetry indices, we analyzed limb‐specific variables (limb stiffness, peak rGRF, change in limb length, and time to peak rGRF) using separate linear mixed‐effects models. These models included treatment group, limb (Surgical vs. Non‐Surgical), and time‐point, along with all two‐way and three‐way interactions among these factors, as fixed effects, with participant treated as a random effect. The inclusion of limb‐level variables was intended to provide a more detailed understanding of the individual limb contributions underlying the observed symmetry measures. These comparisons help to contextualize NSI results by exploring whether changes in symmetry were driven by adaptations in one or both limbs.

Post‐hoc pairwise comparisons were conducted using Tukey's Honestly Significant Difference (HSD) test to further explore significant main and interaction effects. The significance level for all analyses was set at *α* = 0.05. All statistical analyses were performed using JMP Pro 16 (SAS Institute Inc., Cary, NC, USA).

## 3 Results

A total of 33 participants were included in the analysis after excluding four participants in the Biofeedback (BF) group and two participants in the Control (C) group who did not complete post‐intervention and retention assessments, and one participant in the C group was removed from retention visit analysis due to data collection errors. One BF outlier for post‐intervention limb stiffness was removed for exceeding a Mean ± 3*SD threshold. Treatment group demographic details are provided in Table 1.

**Table 1:** Participant information for biofeedback and control groups.

	Biofeedback (*n* = 14)	Control (*n* = 19)	Group comparison
Sex	Male: 8	Male: 10	*p* = 0.815^a^
	Female: 6	Female: 9	
BMI (kg/m^2^)	24.9 ± 4.6	24.2 ± 3.5	*p* = 0.942^b^
Age at Surgery (years)	17.3 ± 1.8	17.4 ± 2.3	*p* = 0.952^c^
Time since Surgery (months)	6.2 ± 1.5	5.6 ± 1.3	*p* = 0.241^c^
ACLR Graft Type	Patellar Tendon: 10	Patellar Tendon: 7	*p* = 0.051^a^
	Hamstring: 4	Hamstring: 12	
Mechanism of Injury	Contact: 5	Contact: 6	*p* = 0.793^a^
	Non‐Contact: 9	Non‐Contact: 13	

^a^ Wald test.

^b^ Wilcoxon Signed‐rank test.

^c^ Independent samples *t*‐test.

**Figure 2:**
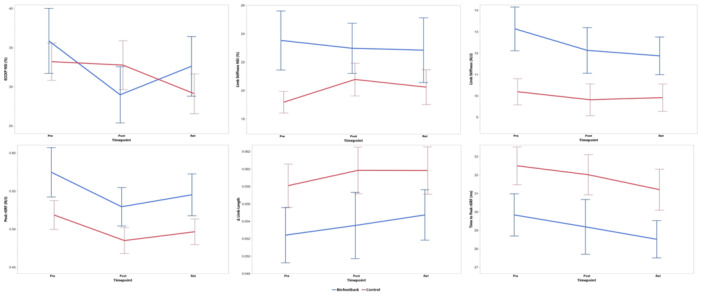
Mean ECCKP NSI, Mean limb stiffness NSI, limb stiffness, peak rGRF, change in limb length and time to peak rGRF with standard error bars at each time point (pre‐intervention, post‐intervention and retention visit). NSI (Normalized Symmetry Index) represent symmetry between the surgical and non‐surgical limbs, while all other metrics are reported for the surgical limb only. (BF: blue, C: red).

For ECCKP NSI, there were no significant treatment group × time‐point interactions (*p* = 0.089). Additionally, no main effects of time‐point (*p* = 0.066) or treatment group (*p* = 0.847) were found. Similarly, limb stiffness NSI did not show a significant interaction between treatment group and time‐point (*p* = 0.694) and no main effects were observed for time‐point (*p* = 0.929) or treatment group (*p* = 0.069) (Figure 2).

For Limb Stiffness, no significant three‐way interaction (limb × treatment group × time‐point) was observed (*p* = 0.979). However, a significant main effect of limb was observed (*p* = 0.001), with the non‐surgical limb demonstrating greater stiffness compared to the surgical limb. No main effect of treatment group (*p* = 0.082) or time‐point (*p* = 0.223) were observed.

For peak rGRF, no significant three‐way interaction (limb × treatment group × time‐point) was observed (*p* = 0.688). A significant main effect of limb was observed (*p* = 0.001), with the non‐surgical limb exhibiting higher rGRF than the surgical limb. Additionally, a main effect of time‐point was noted (*p* = 0.023), with baseline assessments showing higher rGRF compared to post‐assessment values (*p* = 0.014), with no difference baseline and retention assessment (*p* = 0.07). No main effect of treatment group (*p* = 0.157) was observed.

For change in limb length, no significant interaction between limb, treatment group, and time‐point was observed (*p* = 0.774). Moreover, no main effects of time‐point (*p* = 0.514), treatment group (*p* = 0.262), or limb (*p* = 0.145) were found.

For time to peak rGRF, no significant interaction between limb, treatment group, and time‐point was observed (*p* = 0.831). However, a significant main effect for limb was observed (*p* = 0.008), with the non‐surgical limb showing a longer time to reach peak rGRF compared to the surgical limb. No main effect of treatment group (*p* = 0.172) and time‐point (*p* = 0.180) were observed.

## 4 Discussion

This study was a secondary analysis of a pilot clinical trial aimed at decreasing second ACL injury risk using visual and tactile biofeedback and was therefore not powered to detect differences in the secondary analysis outcomes of interest. The current study investigated the effects of a biofeedback intervention on limb stiffness and ECCKP symmetry in patients with an ACLR. However, contrary to our hypothesis, the intervention did not yield significant improvements in ECCKP or limb stiffness symmetry during landing.

Despite expectations that limb stiffness and ECCKP symmetry would improve more in the biofeedback group compared to the control group, no significant interaction between treatment group and time‐point were observed for limb stiffness NSI and ECCKP NSI, suggesting that the intervention did not meaningfully improve interlimb symmetry. These results indicate that a 6‐week biofeedback intervention, when applied to squatting exercises, may not be sufficient to produce meaningful changes in landing mechanics. Traditionally, athletes are cleared to RTS with an LSI greater than or equal to 90% symmetry [49]. While the NSI approach is new, our recent work comparing symmetry metrics in healthy individuals showed poor to moderate agreement between LSI and NSI across functional tasks [50], suggesting that LSI and NSI may reflect different aspects of symmetry, and comparisons between the two should be made cautiously. Although the equivalence between LSI and NSI thresholds remains to be fully established, it is generally assumed that a 10% NSI cutoff corresponds to the 90% LSI benchmark. At the end of the 6‐week trial and retention period, both the groups had average limb stiffness NSI values (BF: 22.9% ± 8.5%, C: 20.2% ± 5.4%) and ECCKP NSI (BF: 32.6% ± 13.7%, C: 29.1% ± 10.8%,) greater than the accepted threshold used when determining an athlete's readiness to RTS. Consistent with these findings, calculated LSI values for both groups remained below the 90% RTS threshold for both limb stiffness (BF: 33.0% ± 22.2%, C: 34.5% ± 22.2%) and ECCKP (BF: 47.5% ± 34.6%, C: 59.7% ± 27.6%). Although this study only analyzed post‐ACLR population, these symmetry findings are concerning, given that athletes are typically cleared to RTS between 9 and 12 months and the average time post‐op at the completion of the study was (BF: 6.2 ± 1.5 months, C: 5.6 ± 1.3 months).

Although the biofeedback intervention did not significantly impact the primary outcomes of interest, the analysis showed a main effect of limb for both limb stiffness (*p* = 0.001) and peak rGRF (*p* = 0.001), with the non‐surgical limb consistently demonstrating greater stiffness and higher rGRF than the surgical limb. The observation that the non‐surgical limb exhibited greater stiffness than the surgical limb may appear counterintuitive; however, it aligns with prior evidence suggesting compensatory offloading of the surgical limb during high‐impact tasks. Findings from Renner et al. demonstrated altered interlimb stiffness symmetry post‐ACLR with the non‐surgical limb stiffness being greater than surgical limb stiffness [51]. Additionally, previous work by Ford et al.has shown that during bilateral landing tasks, the non‐surgical limb often makes ground contact earlier than the surgical limb, suggesting an initial unilateral landing pattern that may further offload the surgical limb in the early phase of impact [52]. In jumping and landing activities, greater limb stiffness corresponds to shorter ground contact times [53], so participants may have preferentially used their non‐surgical limb to land quickly and jump vertically, shortly after initial contact. Although no significant changes in limb stiffness NSI were observed, the consistent finding that the non‐surgical limb exhibited greater stiffness highlights persistent symmetry deficits due to reduced stiffness in the surgical limb (Table 2). This suggests that while NSI captures the degree of symmetry, limb‐wise analysis provides important insight into the direction of the deficit. Together, these findings underscore the need for targeted rehabilitation strategies that directly address mechanical deficits in the surgical limb rather than focusing solely on achieving interlimb symmetry.

**Table 2:** Summary of biofeedback and control group results for surgical and non‐surgical limbs at each time point.

Variable	S/NS	Pre	Post	Ret
Biofeedback Group				
ECCKP NSI (%)		35.86 (14.9)	28.98 (12.9)	32.62 (13.8)
Limb Stiffness NSI (%)		23.50 (7.8)	23.00 (6.6)	22.90 (8.5)
Limb Stiffness (N/J)	S	12.40 (5.8)	10.80 (4.2)	10.90 (3.7)
	NS	13.90 (5.1)	13.30 (6.5)	12.90 (5.4)
Peak rGRF (N/J)	S	0.49 (0.2)	0.48 (0.1)	0.50 (0.1)
	NS	0.65 (0.2)	0.58 (0.2)	0.59 (0.2)
Δ Limb Length	S	0.05 (0.1)	0.05 (0.1)	0.05 (0.1)
	NS	0.05 (0.1)	0.05 (0.1)	0.05 (0.1)
Time to Peak rGRF (ms)	S	29.10 (5.4)	28.90 (7.6)	27.7 (4.0)
	NS	30.60 (6.8)	29.50 (8.4)	29.3 (6.5)
Control Group				
ECCKP NSI (%)		33.23 (10.3)	32.77 (13.2)	29.11 (10.8)
Limb Stiffness NSI (%)		19.20 (3.2)	20.80 (5.0)	20.20 (5.4)
Limb Stiffness (N/J)	S	9.92 (4.2)	9.11 (3.8)	9.40 (3.6)
	NS	10.5 (3.3)	10.5 (5.3)	10.4 (4.3)
Peak rGRF (N/J)	S	0.49 (0.1)	0.45 (0.1)	0.49 (0.1)
	NS	0.55 (0.1)	0.51 (0.1)	0.51 (0.1)
Δ Limb Length	S	0.06 (0.1)	0.06 (0.1)	0.06 (0.1)
	NS	0.06 (0.1)	0.06 (0.1)	0.06 (0.1)
Time to Peak rGRF (ms)	S	31.90 (5.5)	31.20 (6.6)	29.80 (5.5)
	NS	33.10 (7.1)	32.90 (7.0)	32.60 (8.0)

*Note:* “Δ Limb length” represents the within‐trial change in limb segment length during the stop jump task and is not a pre–post change score.

Abbreviations: NS, non‐surgical; NSI, normalized symmetry index; Pre, pre‐intervention; Post, post‐intervention; Ret, retention; S, surgical.

Additionally, a significant main effect of time‐point for peak rGRF (*p* = 0.023) showed that baseline assessments had higher rGRF compared to post‐intervention values. This suggests that, independent of the treatment group, rGRF decreases over time in patients with ACLR, potentially due to ongoing rehabilitation and recovery. However, the non‐surgical limb continued to demonstrate greater loading, supporting previous findings that interlimb symmetry is not fully restored during post‐operative rehabilitation [51, 54, 55].

The time to peak rGRF corresponds with how quickly the limb absorbs the impact of landing and may be influenced by lower extremity muscle strength and neuromuscular coordination [56]. It was expected that the time to reach peak rGRF would be longer in the surgical limb than in the non‐surgical limb considering the observed reliance on the non‐surgical limb when landing. However, the time to peak rGRF was consistently longer for the non‐surgical limb when compared to the surgical limb across the three time points. This contrasts with a previous study that found that the surgical limb took longer to reach peak vertical GRF compared to the non‐dominant limb of uninjured controls [57]. One possible explanation for this discrepancy is that, in our analysis, timing was normalized to the initial contact of each individual limb rather than to the initial contact of the first limb during landing, which may affect interlimb timing comparisons. Prior work by Bates et al. has shown that the choice of timing normalization method can significantly influence the interpretation of landing kinetics and interlimb comparisons in patients with ACLR [58]. Despite the methodological consideration, our results align with previous research indicating that patients with ACLR may adopt altered neuromuscular control strategies, where the surgical limb may demonstrate compensatory mechanisms leading to quicker force application [59]. These persistent symmetry deficits suggest that biofeedback interventions focusing solely on squatting may not adequately transfer to dynamic, high‐impact tasks such as landing.

This study has several limitations. Although our study included 33 participants, post hoc power analysis suggests that we would require approximately 50–80 participants to detect moderate effect size. While our sample was sufficient for large effects, it was likely underpowered for moderate effects, which may explain some of our study results. Larger future studies are needed to confirm these trends and assess the effect of biofeedback on limb stiffness and ECCKP symmetry after ACLR. Secondly, the timeline of standard post‐ACLR rehabilitation may have influenced results. Participants were enrolled 5.6 to 6.2 months post‐surgery, a period when neuromuscular recovery is ongoing [60], which may have limited the additional benefits of a biofeedback intervention beyond standard rehabilitation. The tested intervention required a task transfer from squatting biofeedback to landing assessments. Squatting is a slower, low‐impact movement compared to landing from a jump, and while some studies suggest a relationship between squatting and landing mechanics [61, 62, 63], the effectiveness of this task transfer remains inconclusive. Lastly, the biofeedback group had more participants with patellar tendon grafts compared to the control group. Prior research suggests that patients with patellar tendon grafts exhibit greater knee stiffness and reduced joint displacement during landing [64, 65], which may have influenced the results. Future studies should ensure balanced graft type distribution during randomization to mitigate potential confounding effects.

## 5 Conclusion

Although biofeedback intervention on squatting did not significantly improve interlimb symmetry in KCCKP or limb stiffness during a stop jump, this secondary analysis provides critical methodological and translational insights. These findings demonstrate the feasibility of integrating multimodal biofeedback within rehabilitation settings. Future trials should consider combining the sequential interventions into a single, integrated biofeedback protocol to improve effectiveness and clinical applicability. Additionally, adaptive biofeedback paradigms that evolve with participants' progress may be explored to optimize outcomes. Collectively, these results establish a foundation for refining biofeedback‐driven rehabilitation to restore functional symmetry and support safe return to sport after ACLR.

## Author Contributions

V.R. was responsible for the analysis and interpretation of data, writing/reviewing/editing of the manuscript. B.N.V. was involved in conception, acquisition of data, analysis, and interpretation of data. M.A.T. was involved in conception and research design. S.L.A. was responsible for analysis and interpretation of data, writing/reviewing/editing of the manuscript. R.M.Q. was responsible for conception, analysis, and interpretation of data, writing/reviewing/editing of the manuscript, and providing facilities/equipment. All the authors finally approved the manuscript. R.M.Q. takes responsibility for the integrity of the work as a whole. All authors have read and agreed to the published version of the manuscript.
